# A Case Report of Dialysis Catheter Placement in an Anomalous Pulmonary Vein

**DOI:** 10.1155/crvm/9936069

**Published:** 2025-06-09

**Authors:** Cierra King, Karling Gravenstein

**Affiliations:** Department of Surgery, Memorial Health University Medical Center, Savannah, Georgia, USA

**Keywords:** anomalous venous anatomy, central line placement, venous access

## Abstract

**Background:** Partial anomalous pulmonary venous connections are embryologic defects in which the normal connection between the pulmonary veins and left atrium is disrupted. These rare anomalies are often asymptomatic and identified incidentally. The most common variant is a connection between the left upper pulmonary veins and the left innominate vein. Although typically asymptomatic, these variants are important to be aware of, particularly when performing procedures involving the venous anatomy.

**Case Presentation:** We present the case of a 52-year-old female with a previous history of colon cancer who underwent right hemicolectomy and presented to the hospital due to severe dehydration secondary to profuse nausea, vomiting, and diarrhea. She developed an acute kidney injury with electrolyte derangement and metabolic acidosis requiring initiation of hemodialysis. Due to her preexisting right internal jugular port access, the decision was made to proceed with left internal jugular dialysis catheter access.

Central venous access was performed in standard fashion. There was venous-appearing blood return at the time of needle access and subsequent dilations. However, at the time of catheter advancement, there was noted return of bright red blood and resistance to advancement, concerning for possible arterial cannulation. Concerning arterial placement, an arterial blood gas (ABG) test and chest x-ray were performed; however, the transducer waveforms were not consistent with this. Computed tomography angiography obtained revealed left internal jugular venous access with catheter extension into an anomalous pulmonary vein within the left upper lobe.

The patient was taken to the angiography suite and under fluoroscopy guidance had new left internal jugular catheter access with the catheter terminating successfully in the superior vena cava. She underwent successful dialysis and was subsequently discharged on postprocedure Day 8.

**Conclusions:** Central line placement is a commonly performed procedure in hospitals. There are steps that have been developed to limit complications for this procedure, including ultrasound guidance, visualization of venous blood, and confirmatory imaging prior to use. This is a case in which arterial-appearing blood, paO2, and chest x-ray were concerning for incorrect placement, but additional imaging revealed accurate access with anomalous anatomy. Overall, the case of central line placement in anomalous pulmonary venous connections is rare but needs consideration when the clinical scenario is appropriate.

## 1. Introduction

Partial anomalous pulmonary venous connections are embryologic defects in which the normal connection between the pulmonary veins and left atrium is disrupted. This disruption can arise in a wide spectrum of anatomic variants, including connections involving the superior vena cava, innominate vein, and right atrium [1]. These rare anomalies are often asymptomatic, identified incidentally, and have limited hemodynamic impact [2]. The most common variant identified is the connection between the left upper pulmonary veins and the left innominate vein [1]. Although typically asymptomatic, these variants are important to be aware of, particularly when performing procedures involving the venous anatomy. Central venous line placement is one such procedure, with known complications of arterial injury, pneumothorax, and malpositioning [3].

## 2. Case Presentation

We present the case of a 52-year-old female with a previous history of colon cancer who had undergone right hemicolectomy. Her clinical course had been complicated by local recurrence requiring additional resection and chemotherapy initiation. She presented to the hospital due to severe dehydration secondary to profuse nausea, vomiting, and diarrhea. Due to profound dehydration, she developed an acute kidney injury with electrolyte derangement and metabolic acidosis requiring initiation of hemodialysis. Due to her preexisting right internal jugular port access, the decision was made to proceed with left internal jugular dialysis catheter placement.

**Figure 1:**
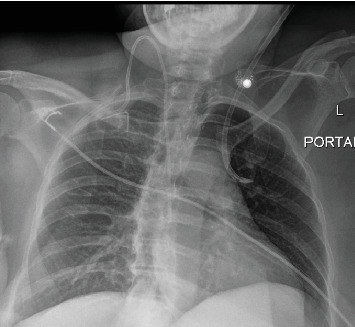
Chest x-ray with concern for catheter malpositioning.

**Figure 2:**
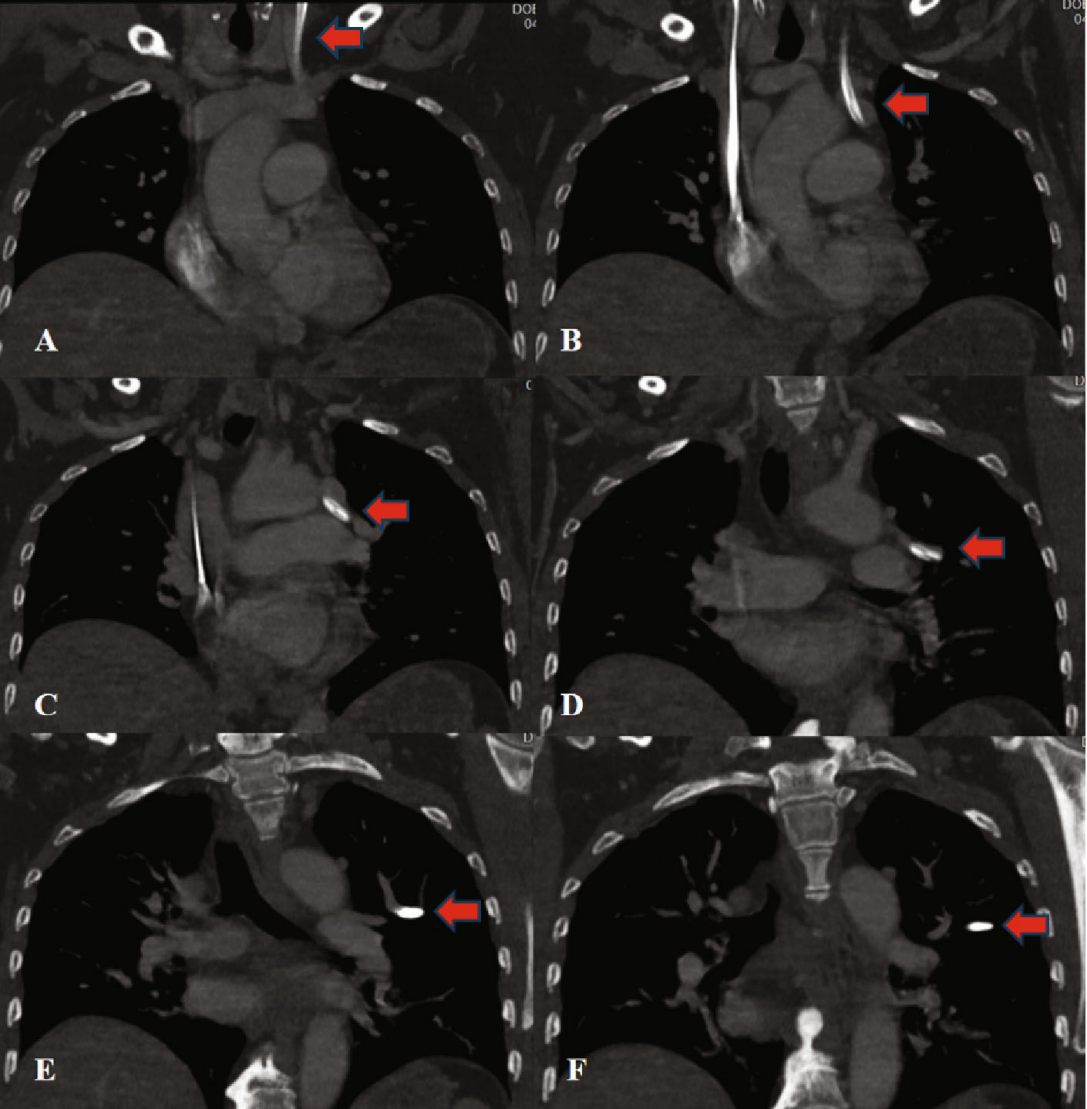
(A) Catheter in left IJ. (B) Catheter crossing left innominate into pulmonary vein. (C) Catheter in pulmonary vein. (D) Catheter extending into pulmonary vein. (E) Catheter within pulmonary vein. (F) Catheter terminating in left upper lobe pulmonary vein.

Central venous access was performed in standard fashion using aseptic technique, with dark venous-appearing blood return at the time of needle access and subsequent dilations. However, at the time of catheter advancement, there was noted return of bright red blood and resistance to advancement concerning for possible arterial cannulation. A blood gas was performed from the line revealing a paO2 of 145, concerning for arterial placement. Chest x-ray was obtained and confirmed malpositioning of the catheter (Figure 1). Pressure transduction revealed venous waveforms. Discussion of concerns for possible intra-arterial catheter placement was held with the patient, and the decision was made to obtain a computed tomography angiography (CTA) for further information and possible operative planning given incongruencies between paO2 and pressure transduction. This CTA revealed left internal jugular venous access with catheter extension into an anomalous pulmonary vein terminating within the left upper lobe (Figure 2).

Subsequently, the patient was taken to the angiography suite. Under moderate sedation and fluoroscopic guidance, a new left internal jugular catheter was placed, with the catheter terminating successfully in the superior vena cava. She underwent successful dialysis and was later discharged on postprocedure Day 8.

## 3. Discussion

Central venous line placement is one of the most commonly performed procedures in the hospital, with around 8% of all hospitalized patients requiring placement [3]. With any high-volume procedure, it is important to consider the complications. In the case of central venous access, these include arterial puncture, pneumothorax, and malposition. Central lines are commonly placed in the subclavian or internal jugular veins, with malpositioning more common in the former due to normal anatomic trajectory. Left internal jugular vein cannulation was more commonly associated with malpositioning than other access sites in a prospective study by Schummer et al. looking at 1794 central line catheterizations [4]. As presented in this article, another less common cause of malpositioning to consider is anomalous anatomy. Beyond considering the possibility, one must be prepared to diagnose and manage said anomalies. Malpositioned internal jugular lines when identified could indicate anomalous anatomy [3].

Central line placement is typically performed in a stepwise fashion to ensure safety. Normally, this involves ultrasound guidance, visualization of venous blood and flow, and confirmatory imaging prior to use. Anomalous anatomy could be identified at any of these steps. In our case, visualization of arterial blood and catheter resistance first sparked concern for malpositioning, reinforced with subsequent imaging. Additionally, we utilized pressure and blood gas analysis to assess our placement. Upon literature review, concern for anomalous anatomy has been identified with each technique (chest x-ray, blood gas, wave form, CT, and fluoroscopy) [5, 6].

As discussed by Alzghoul et al., options for management could include fluoroscopic repositioning or removal, the vast majority being the latter [5, 6]. As in our case, a malpositioned catheter into anomalous pulmonary veins has been successfully managed with repositioning [5]. Overall, the case of central line placement in anomalous pulmonary venous connections is rare, but with the increased risk of malpositioning with left-sided access, there should be consideration of standardized implementation of additional strategies to ensure correct placement and prevent associated complications.

## Data Availability

The authors have nothing to report.

## Nomenclature

paO2: partial pressure of oxygen
CTA: computed tomography angiography
